# Automatic identification and normalization of dosage forms in drug monographs

**DOI:** 10.1186/1472-6947-12-9

**Published:** 2012-02-15

**Authors:** Jiao Li, Zhiyong Lu

**Affiliations:** 1 National Library of Medicine, Bethesda, MD 20894, USA

## Abstract

**Background:**

Each day, millions of health consumers seek drug-related information on the Web. Despite some efforts in linking related resources, drug information is largely scattered in a wide variety of websites of different quality and credibility.

**Methods:**

As a step toward providing users with integrated access to multiple trustworthy drug resources, we aim to develop a method capable of identifying drug's dosage form information in addition to drug name recognition. We developed rules and patterns for identifying dosage forms from different sections of full-text drug monographs, and subsequently normalized them to standardized RxNorm dosage forms.

**Results:**

Our method represents a significant improvement compared with a baseline lookup approach, achieving overall macro-averaged Precision of 80%, Recall of 98%, and F-Measure of 85%.

**Conclusions:**

We successfully developed an automatic approach for drug dosage form identification, which is critical for building links between different drug-related resources.

## Background

Seeking drug-related information is one of the major activities of today's online healthcare professionals and consumers. To date, there are a wide variety of different drug-related resources including but not limited to: the biomedical literature in PubMed^® ^[1], clinical trials in ClinicalTrial.gov [2], adverse drug effects in FDA's Spontaneous Reporting System, and consumer-level drug monographs in MedlinePlus^® ^[3] and PubMed Health [4]. Owing to the heterogeneous nature of each individual resource, they are not currently linked to each other. On the other hand, their contents are often complementary to each other so that users would benefit from an integrated access to all sources relevant to a single drug. Thus, this poses an increasing need to build cross-links between these different resources for the same drug entity so that users from one site can be informed by relevant information in other sites. To this end, a critical step is to be able to identify the drug entity from the corresponding narrative text.

Biomedical named entity recognition (NER) is a challenging task but it serves as a prerequisite for many subsequent tasks like relationship extraction [5]. Over the years, most NER tools have been developed for automatically recognizing gene and gene products from free text using one of the three approaches: dictionary-based, rule-based, and machine-learning based. By contrast, less work involved drug entity identification. Partly, this may be due to the difficulty in defining a drug entity in text. In the earlier work that involved automatic drug entity identification [6-9], a drug was simply defined by its generic name/active ingredient. Such approximation may be appropriate for those applications but to formally define a drug, other important specifications should be considered. For instance, a drug's dosage form (DF) indicates the physical form in which a drug is produced and dispensed. It is one of the most important specifications of a drug because it affects the way a drug is administrated in a patient. Drugs with the same ingredients but in different dosage forms can have different uses. For example, if *timolol *comes as ophthalmic solution (eye drops), it is used to treat glaucoma. If *timolol *comes as an oral tablet, it is used to treat high blood pressure, to improve survival after a heart attack, and to prevent migraine headaches. Furthermore, dosage forms affect drug absorption and drug distribution in the human body. So in order to confirm drug efficacy and optimize drug therapy, drug pharmacokinetic and pharmacodynamic properties should be modeled and experimented across different dosage forms. For example, *tacrolimus*, a macrolide with potent immunosuppressive effects, can come as oral capsule and injectable solution. Its pharmacokinetic properties were studied across intravenous, oral, and intramuscular dosage forms [10]. Because of the importance of dosage form in drug development and consumption, it is crucial to provide accurate dosage form information in drug-related information resources. Therefore, the goal of this work is to automatically identify drug dosage form information from free text and subsequently normalize it using a standardized nomenclature.

Our proposed method is rule-based and is related to two particular areas of previous studies. In the work of clinical drug normalization, Peters *et al*., examined the complexity, ambiguity and variability of clinical drugs (*e.g*., '*Metoprolol Succinate 200 mg sa Tab*') [11]. They processed the clinical name as a string, and defined a set of rules like expanding abbreviations (*e.g., tab to tablet*) to normalize it. However, in their study, dosage form was not segmented from the clinical drug name for further normalization. The other related area is medication information extraction for clinical narratives in electronic medical records (EMRs). Recent studies have focused on extracting both drug names and related attributes such as strength, route, frequency, form, and duration. In 2009, the task of i2b2 challenge was to identify mentions of drugs and drug-related information like dosages and routes of administration from discharge summaries [12]. However, there was no requirement for normalizing mentions to any standardized nomenclature. More recently, several Natural Language Processing (NLP) systems such as MedEx [13] and MTERMS [14] have been developed to automatically normalize identified drugs to concepts in one or multiple terminologies. These research efforts contribute to the field of automated medication reconciliation across the care continuum [15,16]. They successfully applied NLP techniques to summarize and encode the medication data with high performance (F-Measure > 90%). In these systems, mentions of drug dosage form are captured from clinical narratives but not further normalized to a standard controlled vocabulary.

In this study, we present a computational method to identify dosage forms from full-text drug monographs, and normalize them to a standardized nomenclature. Specifically, we used the American Hospital Formulary Service^® ^(AHFS) drug monographs provided by the American Society of Health-system Pharmacists^® ^(ASHP) as our corpus and RxNorm [17] as the standardized nomenclature. To our best knowledge, this is the first work on drug dosage form identification and normalization. For evaluation, we first randomly selected approximately 10% of the AHFS drug monographs and produced human annotations (the gold standard). In addition, to evaluate our method on the entire test data, we further developed a silver standard by automatically extracting the known dosage forms from the drug products that are currently listed in the drug monographs.

## Methods

**Figure 1:**
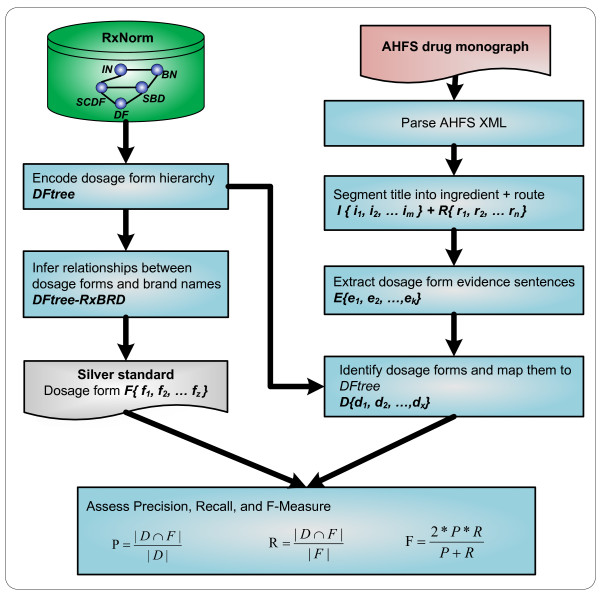
**Workflow for dosage form identification and performance evaluation**. Abbreviations: IN: Ingredients; BN: Brand Name; SCDF: Semantic Clinical Drug Form; SBD: Semantic Branded Drug Form; DF: Dosage Form.

In Figure 1, we show an overview of our working flow for dosage form identification and normalization. We first processed RxNorm by encoding a dosage form hierarchical tree *DFtree *and by establishing dosage form-brand name relationships *DFtree-RxBRD*. Second, we processed each AHFS drug monograph in XML and extracted both the title and sentences mentioning dosage forms in the body of a monograph. From the title and extracted sentences, we identified dosage forms and mapped them to RxNorm concepts. Third, we constructed a silver standard for evaluating the performance of dosage form identification. Finally, we used the *Precision, Recall*, and *F-Measure *as evaluation metrics. The details of each component in our method will be explained in this section.

### Processing RxNorm

RxNorm from the NLM is a standardized nomenclature for clinical drugs and drug delivery devices [17]. In RxNorm, a clinical drug name connects to its ingredients, strengths, and/or dosage forms through a well-defined set of relationships, also known as Term Types (TTY). For example, a clinical drug '*Acetaminophen 500 MG Chewable Tablet*' in RxNorm is connected to its ingredient (TTY = IN) '*Acetaminophen*', its dosage form (TTY = DF) '*Chewable Tablet*', and its brand name (TTY = BN) '*Tylenol*'.

**Figure 2:**
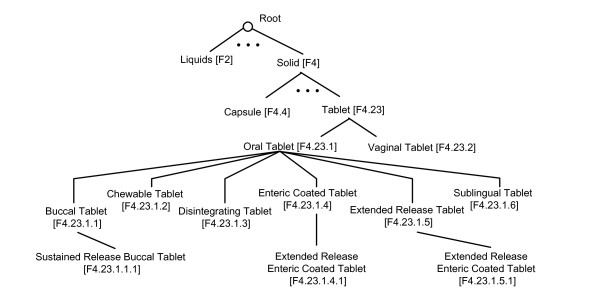
**Part of DFtree: Dosage forms organized in a hierarchical tree structure**.

In this study, we used the RxNorm data in its full monthly release on February 7, 2011. It consists of 100 active dosage forms identified by RxNorm concept identifier (RXCUI). According to their relationship represented in the RxNorm's dosage form definition page [18], we manually encoded and created a hierarchical tree structure called *DFtree *(see complete *DFtree *in Additional File 1). As shown in Figure 2, each dosage form is assigned a specific number to indicate its relative location in the tree. The closer a dosage form is to the root, the more general the concept is. '*F4*' stands for '*Solid*', '*F4.23*' for '*Tablet*', '*F4.23.1*' for '*Oral Tablet*', and '*F4.23.2*' for '*Vaginal Tablet*'. Note that an RxNorm dosage form may be associated with more than one tree number. For example, '*Extended Release Enteric Coated Tablet*' is defined in RxNorm as an enteric coated tablet with a slowed delivery system that allows medication to be released over an extended period of time at a controlled rate. It has two tree numbers '*F4.23.1.4.1*' and '*F4.23.1.5.1*', where the former refers to its relationship to '*F4.23.1.4*' for '*Enteric Coated Tablet*' and the latter to '*F4.23.1.5*' for '*Extended Release Tablet*'. Because of these tree numbers, we were able to automatically determine the specificity of a given dosage form.

### Identifying dosage forms from AHFS drug monographs

AHFS drug monographs represent a tested and proven source of comparative, unbiased, and evidence-based drug information [19]. These full-text monographs, an officially recognized federal standard on drug therapy, have become the major drug information resource of healthcare information systems and public health web sites, such as First DataBank [20], MedlinePlus^® ^[3] and PubMed Health [4]. These monographs are widely viewed and used by healthcare professionals (*e.g*., pharmacists, physicians, and nurses) and consumers.

**Figure 3:**
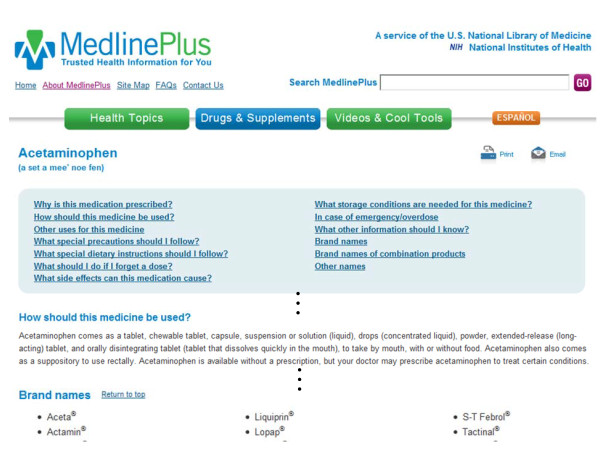
**Snapshot of AHFS drug monograph 'Acetaminophen' in MedlinePlus^®^**.

Each AHFS drug monograph is subject to a drug's generic name/active ingredient (*e.g*., 'acetaminophen') rather than its specific drug product (*e.g*., 'Tylenol^®^'). Each monograph contains comprehensive clinical drug descriptions in separated sections, including 'Why is this medication prescribed?', 'How should this medicine be used?', 'What side effects can this medication cause?', 'Brand names' and etc. Figure 3 shows an example AHFS monograph '*Acetaminophen*' in MedlinePlus^®^, a publicly accessible health-related web site developed by the National Library of Medicine^® ^(NLM). In this study, we collected the most recent 1095 AHFS drug monographs (as of January, 2011). About half (547) of the monographs were devoted for system development and the other half for testing.

To identify dosage forms from the full-text AHFS drug monographs, three sections including 'title', 'How should this medicine be used?' and 'About your treatment' were parsed from the monograph XML files.

#### 1 Segmenting title into ingredient and route

AHFS monograph title indicates the clinical drug discussed in the monograph. By design, the title follows the following pattern:

$$I\left\{i_{1},i_{2},\;\;...i_{m}\right\}+R\left\{r_{1},r_{2},\;\;...r_{n}\right\}$$

Where *I *is a set of ingredients, and *R *is an optional set of administration routes such as '*Topical'*. For example, an ingredient '*Diclofenac*' is discussed in four drug monographs entitled '*Diclofenac*', '*Diclofenac Ophthalmic*', '*Diclofenac Topical*' and '*Diclofenac Transdermal*'. To segment a title into *I *and *R*, we manually created a list of route-indicating terms (*e.g.*, '*nasal*', '*ophthalmic*', '*otic*', '*oral*', '*rectal*', and '*vaginal*') through examining the monograph titles in the development set. These route-indicting terms were used as marker terms. Given a title containing *N *terms *{t_1_, t_2_, ... t_N_}*, if the position of the most left marker term is *q*, then *{t_1_,...t_q-1_} *is segmented as ingredient *I*, and *{t_q_...t _N_} *is as route *R*.

#### 2 Extracting dosage form evidence sentences

The 'How should this medication be used?' Section and 'About your treatment' Section include the detailed descriptions on drug physical forms and its usage. We first segmented the section into individual sentences, and then used a predefined set of patterns (see Table 1) to extract the sentences *E{e_1_, e_2_, ..., e_k_} *that are likely to mention dosage form information. In the five patterns *P1-P5*, both noun and verb variants were allowed. For example, '*drug*' in *P5 *can be 'drugs'; '*come*' in *P1 *and *P2 *can be '*comes*'.

**Table 1:** Patterns for extracting dosage form evidence sentences

Pattern		Description	Example sentences
**P1**	[...] come as [...]	Sentence contains the phrase 'come as' or its variants.	Ophthalmic ciprofloxacin comes as a solution (liquid) and an ointment to apply to the eyes.(from monograph *'Ciprofloxacin Ophthalmic'*)
**P2**	[...] come in [...]	Sentence contains the phrase 'come in' or its variants.	Golimumab injection comes in prefilled syringes and auto-injection devices.(from monograph *'Golimumab injection'*)
**P3**	^[*t_i_*] [...] where, *t_i_∈ I *= {*t_1_*,...*t_q_*_-_*_1_}*	The first term of sentence is an ingredient term in title.	Nicotine gum is used by mouth as a chewing gum and should not be swallowed.(from monograph *'Nicotine gum'*)
**P4**	^[the]medication [...]	The subject of sentence is 'medication' or its variants modified by a determiner.	The medication will be added to an intravenous fluid that will drip through a needle or catheter placed in your vein for 60 minutes two or three times a day.(from monograph *'Quinupristin and Dalfopristin Injection'*)
**P5**	^[the]drug [...]	The subject of sentence is 'drug' or its variants modified by a determiner.	The drug is taken by mouth in capsule form.(from monograph *'Lomustine'*)

Next, to identify RxNorm dosage form from those extracted evidence sentences *E{e_1_, e_2_, ..., e_k_}*, we first added to each sentence *e_j _*with the set of segmented routes *R *from the title, resulting in an enriched sentence set *E^+^{e^+^_1_, e^+^_2_, ..., e^+^_k_}*. An example of such enrichment is shown as below:

**• Title: **Propranolol Oral

**• After title segmentation**: Propranolol [*Ingredient*] Oral [*Route*]

**• Evidence sentence *e_k_*: **It also comes as a solution or concentrate.

**• After appending title route**: It also comes as a solution or concentrate oral

#### 3 Developing route translation rules

We developed a set of translation rules to map popular route expressions in AHFS monographs to terms used in RxNorm *DF *(TTY = DF; *i.e*., RxNorm dosage form concepts). For example, '*by mouth*' was translated to '*oral*', *into nose*' to '*nasal*', '*into ear*' to '*otic*', '*on skin*' to '*topical'*, and '*in eye*' to '*ophthalmic*'. An example of applying such translation rules is shown as below:

**• Title: **Moxifloxacin

**• Evidence sentence *e_k_*: **Moxifloxacin comes as tablet to take by mouth.

**• After applying route translation rules: **Moxifloxacin comes as tablet to take oral.

#### 4 Mapping to RxNorm dosage forms

We turned the evidence sentence after enrichment and translation into a bag of unique words and performed stemming. After separately stemming each word in RxNorm *DFs (e.g. 'oral solution' *to *'oral' and 'solut')*, we looked up individual stems from every RxNorm *DF *in each sentence.

An RxNorm *DF *was identified when all its stems were found in a sentence. In the above '*Propranolol Oral*' example, because of the enrichment with route information from the title, dosage form '*Oral Solution*' was identified. For the other '*Moxifloxacin*' example, '*Oral Tablet*' was able to be identified because of translating '*by mouth*' to '*oral*'.

If more than one *DF *was found in an evidence sentence, we made use of the *DFtree *and kept only the ones that are most specific. For example, a sentence *'Darifenacin comes as an extended-release (long-acting) tablet to take by mouth' *was extracted from the drug monograph titled as '*Darifenacin*'. By translation, '*by mouth*' was changed to '*oral*' so that both '*Oral Tablet*' and '*Extended Release Tablet*' were identified as candidate dosage forms. In this study, we aimed to identify the most specific dosage forms from each drug monograph. As '*Extended Release Tablet*' is a child term of '*Oral Tablet*' in the hierarchical tree (see Figure 2), the latter was subsequently removed and only '*Extended Release Tablet*' was selected.

### Constructing gold and silver standards

To provide a gold standard for evaluation, 100 AHFS drug monographs were randomly selected for manual annotation by two annotators (JL and ZL). 40 monographs were annotated by both annotators with a high inter-annotator agreement [21] of 97.6%.

To assess our method on the entire set of AHFS monographs, we relied on its manually curated drug products (see the bottom part of Figure 3) to construct silver-standard dosage forms, as each drug product is known to be associated with one or more dosage forms in RxNorm. More specifically, the dosage-form silver standard was derived as: we parsed the 'Brand name' Section from each AHFS monograph in XML to extract a list of brand names and subsequently mapped them to RxNorm brand names, each of which was then linked to its dosage form through the semantic branded drug information in RxNorm. For instance, "*Fluoxetine 4 MG/ML Oral Solution [Prozac]*" is a semantic branded drug in RxNorm where both dosage form (oral solution) and brand name (Prozac) are given. By using this method, a set of dosage forms *F{f_1_, f_2_,...f_z_} *were linked to a given drug monograph and was used as the silver standard in this study.

In summary, the gold standard was constructed manually by hand annotating dosage forms in drug monographs, enabling an unbiased evaluation on a subset of test data. On the other hand, the silver standard was constructed automatically from the drug products listed in drug monographs, enabling a comprehensive evaluation on the entire data set.

### Evaluating dosage form identification performance

*Precision, Recall *and *F-Measure *were used as evaluation metrics. Given a drug monograph, *Precision *is the fraction of correctly identified dosage forms over the entire set of identified dosage forms *D*; *Recall *is the fraction of correctly identified dosage forms over the gold-standard/silver-standard dosage form set *F*; and *F-Measure *is the harmonic mean of precision and recall. The overall performance is computed as the macro-average of the three evaluation metrics. In our primary evaluation, for a prediction to be considered as a true positive, it must exactly match the corresponding answer in the gold or silver standard^1^.

$$\begin{array}{c} Precision=\frac{\left|D\cap F\right|}{\left|D\right|};\;Recall=\frac{\left|D\cap F\right|}{\left|F\right|};\\ F-Measure=\frac{2\times Precision\times Recall}{Precision+Recall} \end{array}$$

To evaluate our method performance, we computed all three evaluation metrics against the gold and silver standard respectively. Furthermore, we compared our method with a baseline approach to assess the contribution of the manually developed patterns and translation rules. In the baseline approach, an RxNorm dosage form was identified when all its terms were found in the 'How should this medicine be used?' Section.

## Results

### Result overview

In our experiments, we used the datasets consisting of 1095 AHFS drug monographs (as of January 2011). We randomly divided all 1095 AHFS monographs into two halves, one for development and the other for testing. It is important to note that all the patterns (*e.g.*, for finding evidence sentence) and rules (*e.g.*, for segmenting titles) used in this work were developed based on our examination of the monographs in the development set. That is, the monographs in the test set were used for testing purposes only.

For the 100 annotated drug monographs in the gold standard, 55 were from the development set and 45 from the test set, with an average of 1.55 dosage forms per drug monograph.

**Figure 4:**
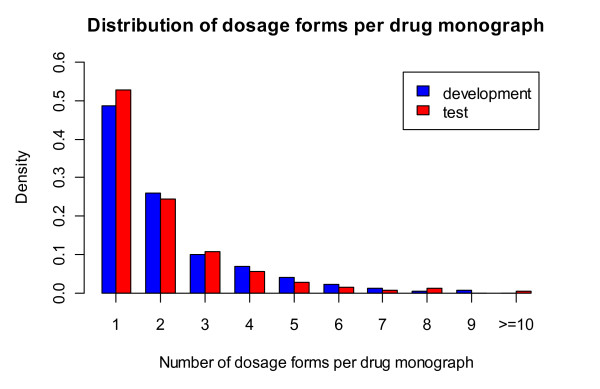
**Comparison of dosage form distribution on development and test set**.

In the silver standard construction, we found 80 dosage forms for 933 monographs among all 1095 monographs. For the remaining 162 monographs that we failed to find their dosage forms, 122 were due to the lack of 'Brand names' Sections (*e.g*., monograph '*Dicloxacillin*'); 20 were due to the failure of brand name mapping (*e.g*., '*Metastron^®^*' in monograph '*Strontium-89 Chloride*'); and 20 were due to missing links between dosage form and brand names in RxNorm (e.g., '*Alphagan P^®^*' in monograph '*Brimonidine Ophthalmic*'). After removing those 162 monographs, we have 462 in development set and 471 in test set. On average, each monograph is linked to 2.09 and 1.97 dosage forms in development and test set, respectively. As shown in Figure 4, the two halves exhibit similar drug dosage forms distributions.

We assessed our identification results against the gold standard and silver standard on both development and test set. Given a drug monograph, *Precision, Recall *and *F-Measure *were calculated. Table 2 shows the results of performance comparison on the mean, standard deviation, median, and interquartile range of these three metrics. As can be seen, the performance of our method on the test set (*F-Measure *of 0.85) resembles that on the development set (*F-Measure *of 0.84), which indicates that the rules and patterns learned from the development set succeeded in the test set.

**Table 2:** Overview of dosage form identification performance

		Gold standard (100)		Silver Standard (933)	
		Development (55)	Test(45)	Development (462)	Test(471)
Precision	Mean	0.81	0.80	0.75	0.70
	Standard deviation	0.30	0.27	0.27	0.31
	Median	1.00	1.00	0.75	0.67
	Interquartile range	0.38	0.50	0.50	0.50
Recall	Mean	0.93	0.98	0.82	0.81
	Standard deviation	0.24	0.09	0.27	0.31
	Median	1.00	1.00	1.00	1.00
	Interquartile range	0.00	0.00	0.40	0.33
F-Measure	Mean	0.84	0.85	0.74	0.71
	Standard deviation	0.27	0.20	0.23	0.27
	Median	1.00	1.00	0.67	0.67
	Interquartile range	0.24	0.33	0.33	0.35

On the test set, our method achieved overall macro-averaged (mean) *Precision *of 80%, *Recall *of 98%, and *F-Measure *of 85% on the gold standard, as well as *Precision *of 70%, *Recall *of 81%, and *F-Measure *of 71% on the silver standard. The decrease in performance on the silver standard is mainly due to its incompleteness and noisiness. Taking the monograph '*Chlordiazepoxide*' as an example, based on the sentence '*Chlordiazepoxide comes as a tablet and capsule to take by mouth*', the two dosage forms '*Oral Tablet*' and '*Oral Capsule*' were manually included to the gold standard for this drug monograph. By contrast, because '*Librium^®^*' is one of the brand names listed in this monograph, its two dosage forms '*Injectable Solution*' and '*Oral Capsule*' were automatically included to the silver standard. As a result, in this case the silver standard misses a true dosage form '*Oral Tablet*' but rather includes a false one '*Injectable Solution*.' Hence, lower system performance is expected on the silver standard than the gold standard.

**Figure 5:**
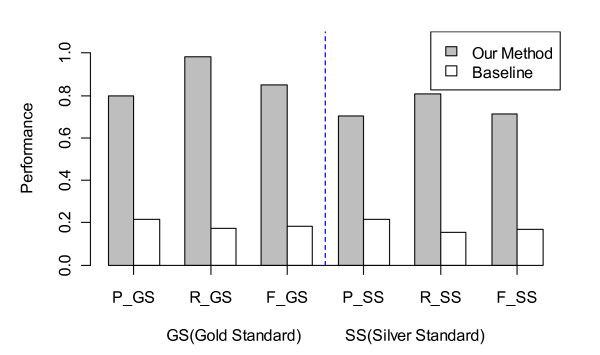
**Comparison of our dosage form identification method with baseline**.

Figure 5 shows the results of performance comparison between our method and the baseline method on the test set. The macro-averaged *Precision, Recall *and *F-Measure *were calculated against both the gold and silver standards. As shown in Figure 5, our method achieved significantly better performance than the baseline method. These results indicate that those manually developed heuristic rules and patterns are important for achieving good performance.

### Error analysis

From our evaluation based on both the gold and silver standards, we can see that our method performed well on the entire monograph collection. Only for very small part (~4%), our method failed to identify any correct dosage forms.

One type of error comes from the fact that we did not take into account a term's relative position in the sentence. For example, '*Cyclobenzaprine comes as a tablet and an extended release capsule to take by mouth*.' is the dosage form evidence sentence extracted from monograph '*Cyclobenzaprine*'. As our method represented it as a bag of words, in addition to the correct dosage form '*Extended Release Capsule*', an incorrect DF '*Extended Release Tablet*' was also identified. Hence, the *Precision, Recall *and *F-Measure *decreased to 50% in this case.

For those monographs where we failed to identify any correct dosage forms, one main reason is because there is neither 'How should this medication be used?' nor 'About your treatment' Section in these monographs (*e.g.*, '*Polio Vaccine*' and '*Typhoid Vaccine*'). Thus, our method failed to identify any evidence sections or sentences from these monographs. As a result, empty results were returned by our method in these cases.

## Discussion

### Practical implications of this research

We successfully developed an automatic method to identify dosage forms from full-length AHFS drug monographs and further normalize them to RxNorm concepts. By doing so, our method can contribute to several real world applications with regards to the AHFS drug monographs and its related resources:

1 Through RxNorm, our method can automatically retrieve other drug attributes and further link a pre-identified drug to other medical terminologies. For instance, after normalizing a drug mention to RxNorm based on its ingredient and dosage form, one can easily retrieve more drug attributes in RxNorm such as National Drug Code (NDC) and information on drug prescription and human/veterinarian use. Additionally, RxNorm is part of the Unified Medical Language System^® ^(UMLS^®^)[22] which brings together many health and biomedical vocabularies and standards (134 sources in 2011AA release) to enable interoperability between computer systems. If a drug has an RxNorm identifier, it can be further linked to other resources in UMLS such as National Drug File-Reference Terminology (NDF-RT) [23] and Medical Subject Headings (MeSH) [24].

2 Through RxNorm, our method can help AHFS editors update and revise their drug page information. Because RxNorm is updated on a regular basis, our method allows to use the latest RxNorm to identify any new or different information about a drug in an AHFS page. Taking the drug '*Vardenafil*' for example, we were able to identify '*Oral Tablet*' from its full-text monograph. Through RxNorm, we found two drug products ("*Levitra*" and "*Staxyn*") both containing active ingredient '*Vardenafil*' and made as oral tablet. However, the drug brand name "*Staxyn*" is currently missing in its corresponding monograph due to the fact that it is a new drug approved in June, 2010 [25], while the '*Vardenafil*' monograph was last updated in September, 2008.

3 Our method can help more accurately link drug monographs to other related resources. At present, the AHFS monographs in MedlinePlus have been linked with other NLM resources such as ClinicalTrial.gov [2] and NLM Drug Information Portal [26] through ingredients. However, drugs with the same ingredient but in different dosages forms may need to be distinguished and linked differently. For example, there are four *Diclofenac *related monographs in MedlinePlus (titled as '*Diclofenac*', '*Diclofenac Transdermal*', '*Diclofenac Ophthalmic*', and '*Diclofenac Topical*' respectively). In this case, instead of linking them to the same set of clinical trials, our method enables us to link them to different trials where drugs of specific dosage forms were studied.

### Limitations of our study

This study has two limitations. First, although our rule-based method has shown robust and quite satisfactory results on the blinded test set, it was specifically developed for finding dosage forms in the AHFS drug monographs. For example, the titles of AHFS drug monographs followed the "ingredient + route" pattern. Therefore, when applied to other types of documents, extra patterns or rules may be needed.

Second, to evaluate method performance on the entire set, we constructed a silver standard based on the assigned drug products. Although this computed "silver standard" enabled us to perform a feasible large-scale evaluation, its own accuracy (as shown in the 'Result overview' Section) may underestimate the performance of our method.

## Conclusions

In this study, we presented a rule-based method to automatically identify and normalize a medication's dosage forms to a standardized nomenclature (*i.e*., RxNorm dosage forms). Our rules were independently developed on the development set. For evaluation, we manually annotated dosage forms for ~10% of drug monographs (the gold standard), on which our method achieved macro-averaged *Precision *of 80%, *Recall *of 98% and *F-Measure *of 85%. In addition, for a larger scale evaluation on the entire test data, we derived a silver standard based on the known drug products associated with the drug monographs. Accordingly, our method achieved *Precision *of 70%, *Recall *of 81% and *F-Measure *of 71%. As discussed, the performance numbers on the silver standard are likely to be underestimated due to the quality of the silver standard.

Our method has been successfully applied to a large set of drug monographs as a step toward providing users with integrated access to multiple drug-related resources. It may also be helpful for monograph editors to revise and update drug information for improving the quality of consumer health information on medications. Finally, our results illustrate that the rules and patterns developed in this work are critical for achieving good performance. We expect our method to be beneficial to applications with regards to the AHFS drug monographs and its related resources.

## Endnotes

^1^No significant difference in evaluation results was found when considering the hierarchical relationships between dosage forms when comparing our predictions with the answers.

## Competing interests

The authors declare that they have no competing interests.

## Authors' contributions

JL and ZL conceived the whole study, participated in its design, analyzed the results and wrote the manuscript. JL implemented methods and performed the experiments. All authors read and approved the final manuscript.

## Pre-publication history

The pre-publication history for this paper can be accessed here:

http://www.biomedcentral.com/1472-6947/12/9/prepub

## Supplementary Material

Additional file 1 **A complete set of *DFtree *information**. Click here for file
